# Comparison of Patient-Reported Experience of Patients Receiving Radiotherapy Measured by Two Validated Surveys

**DOI:** 10.3390/curroncol28030202

**Published:** 2021-06-12

**Authors:** Abdulla Al-Rashdan, Linda Watson, Demetra Yannitsos, Siwei Qi, Petra Grendarova, Lisa Barbera

**Affiliations:** 1 Tom Baker Cancer Centre, Calgary, AB T2N-4N2, Canada; Abdullah.Al-rashdan@ahs.ca (A.A.-R.); demetra.yannitsos@ahs.ca (D.Y.); 2 Cumming School of Medicine, University of Calgary, Calgary, AB T2N-1N4, Canada; 3 Applied Research and Patient Experience, Cancer Research and Analytics, Cancer Care Alberta—Alberta Health Services, Calgary, AB T2S-3C3, Canada; Linda.Watson@ahs.ca (L.W.); siwei.qi@ahs.ca (S.Q.); 4 Grande Prairie Cancer Centre, Grande Prairie, AB T8V-2E8, Canada; petra.grendarova@ahs.ca

**Keywords:** ambulatory oncology patient satisfaction survey, AOPSS, Your Voice Matters, YVM oncology, ambulatory, cancer, patient-reported experience, person-centred care, patient-centred care

## Abstract

Patient-reported experience is associated with improved patient safety and clinical outcomes. Quality improvement programs rely on validated patient-reported experience measures (PREMs) to design projects. This descriptive study compares the experience of cancer patients treated with radiation as recorded through the Ambulatory Oncology Patient Satisfaction Survey (AOPSS) or as recorded through Your Voice Matters (YVM) between February and August 2019. Six questions were compared (“overall experience with care”, “discussion of worries”, “involvement in decisions”, “trusting providers with confidential information”, “providing family with information”, and “knowing who to contact”). Positive experience scores were calculated by cohort and by tumor groups. Multivariable logistic regression models evaluated factors associated with positive experience. Two cohorts (220 and 200 patients) met the eligibility criteria for the AOPSS and YVM, respectively. Positive experience was reported similarly between the two PREMs for “overall experience with care”, “discussion of worries”, and “trusting providers with confidential information” with a score difference of 1–4% at the cohort level. Positive experience score difference ranged from 5% to 44% across questions at the tumor group level. Different experience gaps were identified with the two measures, mainly at the tumor group level. Programs interested in using these PREMS might consider this when designing projects.

## 1. Introduction

Patient-reported experience is an important indicator of quality of care. Better patient experience is associated with improved patient safety, clinical outcomes, and healthcare resource utilization [1,2,3,4,5]. The Beryl institute defines patient-reported experience as the sum of interactions, shaped by an organization’s culture, that influence patient perceptions across the continuum of care [6]. Given that quality of care is not only assessed by success in the technical aspects of medical procedures but also by measuring the way that healthcare services were delivered, there has been growing interest in including experience measures in quality improvement (QI) initiatives [7,8,9,10,11].

Patient-reported experience measures (PREMs) are tools that collect information on patients’ views of their experience during their treatment journey [12]. Several validated PREMs are used in oncology outpatient settings [13,14]. The Ambulatory Oncology Patient Satisfaction Survey (AOPSS) was developed by the Picker Institute and has been utilized in many Canadian provinces since 2005 [13]. It retrospectively captures the experience of patients who received active outpatient oncology treatment within the previous six months. Your Voice Matters (YVM) was developed by Cancer Care Ontario specifically for ambulatory oncology patients and has been used routinely in Ontario for the past several years [14]. It is used in real time and measures experience specific to an outpatient encounter.

Oncology programs aiming to improve patient experience rely on validated PREMs to identify gaps and determine which areas are in need of improvement [15,16,17,18,19,20,21,22]. One such program is the Person-centered Radiation Oncology Service Enhancement (PROSE) [23], which is a programmatic initiative whose goal is to develop person-centered care in the radiation department of our cancer center through targeted projects aimed at improving patient experience and quality of care. As preliminary work, the program sought to understand how AOPSS and YVM might guide our work. The aim of this project is to compare and contrast experiences measured by these two PREMs both for a large sample and then specifically by tumor group.

## 2. Methods

### 2.1. Study Design and Participants

We conducted a descriptive study comparing results from the AOPSS and the YVM. The AOPSS is administered by mail to eligible patients in Alberta every two years as part of a regular organizational standard. The PROSE program prospectively administered the YVM to record the experience of patients treated with radiotherapy in real time. Although both PREMs assessed experience within a short time-frame for respondents treated in the same institution, there was no overlap between the two surveyed cohorts. The differences between the uses of the two PREMs can be seen in Table 1.

Eligibility criteria included respondents aged 18 years or older with any confirmed malignant tumor, who had the ability to read and write in English, and who agreed to fill out the PREM. For the AOPSS, only respondents who indicated that they received radiation treatment in our institution were included in this study.

### 2.2. Patient-Reported Experience Measures

The AOPSS is a validated paper-based PREM used in multiple jurisdictions in Canada and internationally. The AOPSS was mailed in February 2019 to a random sample of Albertan patients who had a cancer diagnosis and had received at least one ambulatory cancer treatment in the previous six months. Cancer treatment was classified as any systemic or oral therapy (e.g., chemotherapy or immunotherapy) and/or radiotherapy. Patients completed the survey at home and returned it via a prepaid envelope. The National Research Corporation (NRC) Health–Canada collected the results and returned them to the provincial cancer agency. The AOPSS consists of 97 questions that retrospectively assesses whether the various patient experience aspects were met from diagnosis to follow-up including the period when they received their care. Completed surveys can reflect the experience of patients recorded weeks to months after their interaction with the healthcare system. The AOPSS experience questions included in this study have three to six possible response options with the most common response options being: yes, yes somewhat, and no.

The YVM is a validated PREM that is used routinely throughout the province of Ontario. The YVM was completed on a tablet, in real time, while the patient was present in the radiation department. The research assistant approached consecutive patients undergoing radiation treatment between May–August 2019 and the aim was to recruit equal proportions of patients by tumor sites to establish baseline experience. The YVM consists of 28 main questions with sub-questions that assess several patient experience aspects, including the patients’ arrival to the clinic, during their appointment, and their departure. All experience questions analyzed in this study had six response options (excellent, very good, good, fair, poor, and not applicable). The completed responses were stored on a REDCap secured server.

### 2.3. Outcome Definition

The primary outcome was to compare reported experience for each cohort. Questions that were similar in content and wording between the PREMs and reflected important experience aspects were identified by four authors (AA, PG, LB, and DY), Table 2. Positive experience scores were calculated and compared between the two cohorts. An important experience indicator is the “overall experience with care” which had similar response options in both PREMs. Positive experience scores were calculated as the number of “excellent” and “very good” responses over the number of all valid responses. The remaining experience questions were “discussion of worries”, “involvement in decisions”, “trusting providers with confidential information”, “providing family with information”, and “knowing who to contact”. For these questions, positive experience scores were calculated in AOPSS as per the NRC method as the number of single positive responses over the total number of valid responses [13]. For YVM it was defined as the number of “excellent” and “very good” responses over the total number of valid responses. Questions that were not answered or had a “not applicable” answer were not considered valid and were excluded from the denominator of the calculation of for both PREMs. Positive experience scores were also calculated at the corresponding tumor group level for each of the selected questions.

### 2.4. Statistical Analyses

The AOPSS and YVM cohorts were characterized using descriptive statistics. Covariates included those common to both PREMs: age, sex, education, and tumor group. Tumor group was defined as breast, lung, gastrointestinal, genitourinary, and other. Chi-Square tests of independence were performed to examine if the outcomes differed between the two cohorts. Logistic regression evaluated the association between dichotomized positive patient experience (yes/no) and every variable of the demographic/clinical factors within each cohort. Model fit was assessed by Hosmer–Lemeshow goodness-of-fit tests. In the AOPSS cohort, the model could not generate a parameter estimate for “trusting providers with confidential information” and “knowing who to contact”. However, this did not affect the calculation for other variables in the model. Data were exported into SPSS Version 25.0 (Chicago, IL, USA) for analysis and statistical significance was set a priori at *p* < 0.05.

## 3. Results

### 3.1. Sample

The AOPSS was sent to a random sample of 1200 patients. A total of 584 completed and returned the survey, resulting in a 49% response rate. Of the 584 respondents, 220 had received radiotherapy in our institution and therefore met the eligibility criteria to be included in our study. A total of 257 patients in the same radiotherapy department were approached to complete the YVM and 200 patients completed it resulting in a response rate of 78%.

### 3.2. Patient Characteristics

Around half of the respondents (52%) were between the ages of 50–70 for both PREMs. Females represented 58% of all respondents in the AOPSS group compared with 52% in the YVM group. Almost equal proportion of respondents to both PREMs completed a college, trade, or technical school (29% and 27% respectively), and those who completed up to high school degree were 37% for the AOPSS and 31% in the YVM. There were no statistical differences in the distribution of age, sex, or education between the two cohorts. There was a significant difference in distribution of tumor groups with breast cancer comprising 33% of the AOPSS respondents compared to 14% of the YVM respondents. The other group comprised 27% of AOPSS respondents in comparison to 41% of the YVM respondents, *p* < 0.001, (Table 3).

### 3.3. Positive Experience at the Cohort Level

**Figure 1:**
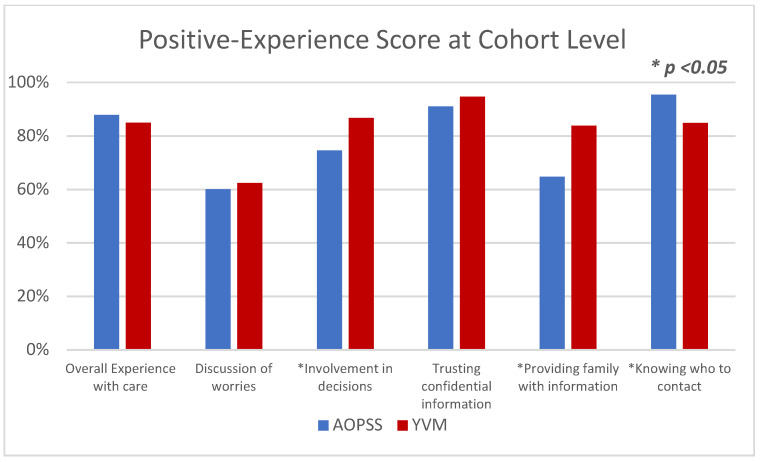
Patient-Reported Experience for the AOPSS and YVM Cohorts. AOPSS, Ambulatory Oncology Patient Satisfaction Survey; YVM, Your Voice Matters. * Represent a statistically significant difference.

Positive experience scores were reported similarly at the cohort level with minimal score differences (1–4%) between the two PREMs for three of the six questions (Figure 1). The positive “overall experience with care” score was 87.9% for the AOPSS cohort and 85% for the YVM cohort and both cohorts had similar positive experience scores in “discussion of worries” and “trusting provider with confidential information”. The YVM cohort reported higher positive experience scores in “involvement of decisions” and “providing family with information”, while the AOPSS cohort reported a higher positive experience score than the YVM cohort in “knowing who to contact”.

### 3.4. Positive Experience at the Tumor Groups Level

**Figure 2:**
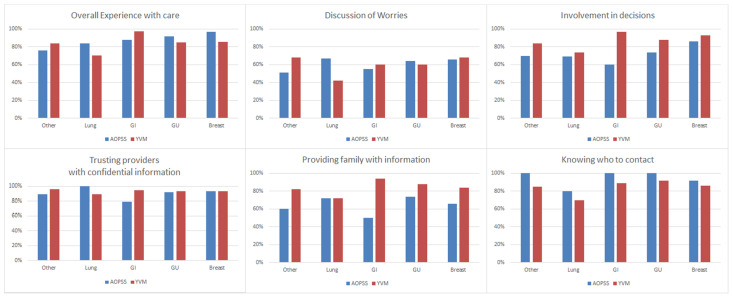
Differences in Positive Experience of AOPSS to YVM by Tumor Group. AOPSS, Ambulatory Oncology Patient Satisfaction Survey; YVM, Your Voice Matters; GI, Gastrointestinal; and GU, Genitourinary.

Differences in positive responses for each question, by tumor group are presented in Figure 2. Positive experience scores by tumor group differed in all questions. The “overall experience with care” was reported more favorably in AOPSS in three tumor groups (lung, breast, and genitourinary). The largest score difference was observed in the lung group (14%). Conversely, overall experience of care was reported more favorably in YVM in two tumor groups (gastrointestinal and other) with the largest score difference observed in the gastrointestinal group (9%).

In the “discussion of worries”, respondents to AOPSS lung group reported more favorable experience than the YVM lung group (67% vs. 42%, respectively). For “involvement in decisions”, AOPSS respondents indicated a less favorable experience in all tumor groups in comparison to the YVM respondents, with the largest difference observed in the gastrointestinal group (60% vs. 97%, respectively). For “trusting providers with confidential information”, AOPSS respondents with a gastrointestinal cancer indicated a less favorable experience compared to YVM respondents (79% vs. 95%, respectively) whereas the lung cancer respondents in the AOPSS reported more favorable experience in comparison to the YVM respondents (100% vs. 89%, respectively). For “providing family with information” a less positive experience was observed for AOPSS respondents in all tumor groups, except lung with the largest difference was in the observed in the gastrointestinal group (50% vs. 94%, respectively). For “knowing who to contact”, a more positive experience was observed for AOPSS respondents in all tumor groups in comparison to the YVM respondents.

### 3.5. Association between Positive Experience and the Covariates within Each Cohort

Multivariable analysis was conducted for each cohort separately to assess the association of each of the covariates with a positive experience. The results of Hosmer–Lemeshow tests revealed the data fit the 12 models well, with all the alphas above 0.05 (minimum = 0.125). Age and sex were not associated with experience for either cohort for any of the experience questions. Education level was a significant factor for both PREMs in “discussion of worries”, with patients with a college degree more likely to report a positive experience compared to those who only completed up to high school (Odds Ratio (OR) 2.4, 3.2, respectively). A similar association was observed for “trusting providers with confidential information” in the AOPSS respondents (OR = 5.6, 95% Confidence Interval (CI) 1.1–28.4). Certain tumor groups were also associated with positive experience in the YVM cohort; respondents from the breast group were more likely to report a positive experience when compared to the lung group within YVM respondents, when asked about “involvement in decisions” (OR = 6.7, CI 1.04–43). Respondents from the gastrointestinal group were more likely to report a positive experience when compared to the lung group in “overall experience with care” (OR = 11, CI 1.2–103), “involvement in decisions” (OR = 14.2, CI 1.5–136), “providing family with information” (OR = 6.8, CI 1.1–41) and “knowing who to contact”. In the AOPSS, there was no significant association between tumor groups and experience (Table 4).

## 4. Discussion

When comparing experience as recorded by AOPSS and YVM, we observed that positive experience was reported similarly for three out of the six selected experience questions on the cohort level, but notable differences existed by tumor groups. The relative similarities of experience at the cohort level and differences at the tumor group level suggests that programs may need to carefully consider which PREM meets their needs best and that it may be necessary to evaluate smaller sub-groups to find actionable gaps in experience and effectively guide QI projects.

To the best of our knowledge, this is the first study comparing the results of these two PREMs. This comparison is not meant to suggest that one PREM is better than the other in assessing experience but is meant to inform healthcare professionals and QI programs that patient experience might be different when recorded by these two PREMs. The AOPSS might be used at an institutional level and help guide systematic changes that will reflect global evaluation of experience. Comparatively, the YVM, which captures experience at point-of-care, might be better suited for developing QI projects at the departmental level but may not capture a global perspective of experience.

Our study evaluated similar experience aspects recorded by two PREMs for patients treated in the same institution, and we observed differing results by tumor group. Had we used the AOPSS only, we might have developed a QI project to address the less favorable experience seen in the gastrointestinal group that had lower positive experience scores in comparison to the YVM gastrointestinal group resulting in differences in experience aspects ranging from 5% to 44%. Had we used the YVM, we might have chosen to target the lung group instead. This illustrates the importance role of context when interpreting the results from these two measures.

One reason for difference in reported experience might be because of the timing of recording of experience. The AOPSS records experience at a later time-point compared to the YVM. Patients continue to process what has happened to them (cognitively, emotionally, and psychologically) [24] during and after their interaction with the healthcare system, and recording experience whilst receiving care might yield different results compared to recording it after the interaction ends where the clinical outcome might be known to patients and their dependency on the healthcare system is less. Further, there may be recall bias and inaccuracies for answering some experience questions after a longer period from the interaction with the healthcare system [25,26]. Real-time PREM reflect patient views regarding a particular encounter and are less prone to recall inaccuracies. However, they may not capture the full experience that patients develop over a longer period as their views change. Previous studies looking at the time for recording experience in patients discharged after in-hospital stay have suggested that patients reported worse experience more often when the survey was administered at a longer period after discharge [27,28,29,30,31,32]. For example, in a large nationwide study, Bjertnaes [27] showed that patients had the worst experience on three out of six scales when the survey was conducted further from the date of discharge from hospital. Finally, differences in timing may affect the patients included in the sample. In our study, a less favorable experience was recorded by AOPSS for all tumor groups except lung, which reported a more favorable experience. Since APOSS is administered sometime after the care is completed, patients with a poor prognosis may not survive or be too ill to respond. So, it is likely that the lung respondents to APOSS have already experienced a better clinical outcome which influences their experience. Patients who are actively receiving care at the cancer centre may be sicker and more likely to be included by a measurement made in real time (like YVM).

The limitations of this study include our inability to compare experience recorded by the two PREMs on the same cohort of patients which may limit our conclusions. Also, there was different distribution of tumor groups between the two cohorts and some tumor groups might be affected by factors external to the cancer system (e.g., support for breast cancer). However, we attempted to mitigate any confounding from tumor group differences by including it as a variable in the model. Another limitation is the small sample size for respondents in certain tumor groups for the AOPSS which limits the power of our model. However, this represented the sample of the respondents from our department for that year and it was similar to the sample size we received in 2017. Also, we were limited to the variables that were collected in both PREMs and there were other variables that may be associated with experience, such as treatment intent [33], that were not available from the YVM to compare. Finally, there are various aspects to experience that the two PREMs assess which we could not directly compare as we only selected the most similar experience questions. For example, although both PREMs assess if providers discussed worries and concerns, the AOPSS asks if a referral was made to help patients with their worries and concerns while the YVM does not.

## 5. Conclusions

Patient-reported experience gaps may differ when measured by AOPSS and YVM on the cohort level and this was more pronounced at the tumor group level. Programs that design QI initiatives to improve experience need to consider this in the context of the strength and limitations of these PREMs when identifying gaps in experience.

## Figures and Tables

**Table 1:** Differences between the two PREMs.

Items	AOPSS	YVM
Data collection	Retrospective	Real time
Location where the PREM was completed	Home	Hospital
PREM type	Paper	Electronic
Assessment of experience over a period of	within six months	within the last outpatient visit (recorded in real time)
Healthcare services assessed	Multiple services that the patient might have interacted with including radiotherapy	One service (radiotherapy)
Number of questions in the PREM	97 questions	28 main questions with some having sub-questions

PREM, Patient-Reported Experience Measure; AOPSS, Ambulatory Oncology Patient Satisfaction survey; and YVM, Your Voice Matters survey.

**Table 2:** Experience questions that were compared between the AOPSS and the YVM.

Items	AOPSS	YVM
OEDW	Overall, how would you rate your quality of care in the past six months? If you had any worries or concerns before beginning your treatment, did your care provider discuss them with you?	On a scale of excellent to poor, how would you rate the following…? Your overall experience with your last visit? Please think of one of the providers you met with today and identified in Question 13. On a scale of excellent to poor, how would you rate this doctor/healthcare provider on the following…? Discussed your emotional worries and concerns?
ID	Were you involved in decisions about your care as much as you wanted?	Please think of one of the providers you met with today and identified in Question 13. On a scale of excellent to poor, how would you rate this doctor/healthcare provider on the following…? Involved you in decisions about your care in the way you wanted?
TC	Did you feel you could trust your care providers with confidential information?	On a scale of excellent to poor, how would you rate the following….? Your overall confidence that the people in the clinic treat your health information with privacy/confidentiality?
PI KC	Did your care providers give your family or someone close to you all the information they needed to support you in your care and recovery? Did you know whom to talk to when you had any questions or concerns?	On a scale of excellent to poor, how would you rate the following….? Your overall confidence that the clinic provided your family/caregiver with the information they wanted about your care? On a scale of excellent to poor, how would you rate the following….? Your overall confidence that the clinic ensured that you know who to contact if you have any questions or concerns after your most recent visit?

PREM, Patient-Reported Experience Measure; AOPSS, Ambulatory Oncology Patient Satisfaction Survey; YVM, Your Voice Matters; OE, Overall experience with care; DW, Discussion of worries; ID, Involvement in decisions; TC, Trusting providers with confidential information; PI, Providing family with information; and KC, Knowing who to contact.

**Table 3:** Participant Characteristics.

Items	Surveyed Cohorts		
	AOPSS (N = 220)	YVM (N = 200)	*p*
Age groups <50 51–70 ≥71	31 (14%) 114 (52%) 75 (34%)	41 (21%) 104 (52%) 51 (26%)	0.080
Sex Female Male	128 (58%) 92 (42%)	103 (52%) 97 (49%)	0.169
Education levels Up to high school graduate College, trade, or technical school University degree and/or more	81 (37%) 64 (29%) 62 (28%)	62 (31%) 54 (27%) 66 (33%)	0.387
Tumor groups Breast Genitourinary Gastrointestinal Lung Other	72 (33%) 38 (17%) 25 (11%) 26 (12%) 59 (27%)	28 (14%) 27 (14%) 37 (19%) 27 (14%) 81 (41%)	<0.001

AOPSS, Ambulatory Oncology Patient Satisfaction Survey; and YVM, Your Voice Matters.

**Table 4:** Results of Multivariable Logistic Regression Model for Similar Experience Items from AOPSS and YVM.

Items		Overall Experience with Care		Discussion of Worries		Involvement in Decisions		Trusting Providers with Confidential Information		Providing Family with Information		Knowing Who to Contact	
	Reference Level	AOPSS OR (95% CI)	YVM OR (95% CI)	AOPSS OR (95% CI)	YVM OR (95% CI)	AOPSS OR (95% CI)	YVM OR (95% CI)	AOPSS OR (95% CI)	YVM OR (95% CI)	AOPSS OR (95% CI)	YVM OR (95% CI)	AOPSS OR (95% CI)	YVM OR (95% CI)
**Age group**													
<50	≥71	0.5 (0.1–2.2)	0.6 (0.2–2.7)	1.6 (0.6–4.3)	0.3 (0.1–1.2)	0.5 (0.2–1.4)	0.4 (0.1–2.4)	0.2 (0.03–1.2)	0.5 (0.05–4.6)	0.8 (0.3–2.1)	0.5 (0.1–2.2)	NA	0.37
51–70		0.9 (0.3–2.6)	0.7 (0.2–2.2)	1.7 (0.8–3.6)	0.7 (0.2–2)	0.9 (0.4–1.9)	0.4 (0.1–1.6)	0.4 (0.1–1.6)	0.4 (0.7–2.3)	0.7 (0.3–1.5)	0.7 (0.2–2.4)	2 (0.1–35)	0.38
**Sex**													
Female	Male	1.4 (0.5–4.3)	0.97 (0.3–2.8)	2.3 (0.3–6.4)	1.1 (0.5–2.7)	0.8 (0.3–2.1)	0.6 (0.2–1.9)	0.3 (0.03–1.9)	1.1 (0.2–5.1)	0.7 (0.3–1.7)	0.7 (0.2–1.9)	NA	0.5 (0.2–1.6)
**Education level**													
College	Up to high school	2 (0.6–6.9)	2.5 (0.8–8.2)	**2.4** **(1.04–5.3)**	**3.2** **(1.1–9.1)**	1.97 (0.9–4.5)	2 (0.5–7.5)	**5.6** **(1.1–28.4)**	1.2 (0.2–6.5)	1.6 (0.7–3.5)	2 (0.6–7.1)	NA	1.6 (0.4–6.8)
University or more		1.1 (0.4–3.4)	2.96 (0.9–9.7)	0.99 (0.4–2.2)	2.3 (0.7–6.7)	1.8 (0.8–4.2)	1.8 (0.5–6.2)	3.1 (0.9–11.2)	1.8 (0.4–9.5)	0.7 (0.3–1.5)	2.5 (0.7–8.6)	NA	0.7 (0.2–2.4)
**Tumor group**													
Breast	Lung	4.98 (0.7–38)	3.0 (0.6–16.1)	0.6 (0.1–2.3)	4.3 (0.9–19.5)	3.3 (0.9–11.8)	**6.7** **(1.04–43)**	NA	2.1 (0.2–17.1)	0.98 (0.3–3.3)	5.9 (0.9–37.6)	NA	**5.6** **(1.01–31)**
GI		1.4 (0.2–8.1)	**11** **(1.2–103)**	0.7 (0.2–2.9)	1.5 (0.4–5.9)	0.6 (0.2–2)	**14.2** **(1.5–136)**	NA	2.8 (0.4–19.9)	0.2 (0.1–1.5)	**6.8** **(1.1–41)**	NA	**6.2** **(1.2–31.5)**
GU		1.99 (0.3–12.2)	2.1 (0.4–12)	1.6 (0.3–5)	1.5 (0.3–8)	0.9 (0.2–3.1)	3.2 (0.5–21.9)	NA	4.4 (0.3–59.9)	0.94 (0.2–3.7)	3 (0.5–21)	NA	7.03 (0.6–77.6)
Other		0.6 (0.1–2.6)	1.4 (0.4–5.1)	0.4 (0.1–1.4)	2.4 (0.7–8.8)	1.2 (0.4–3.7)	2.2 (0.6–8.2)	NA	4 (0.6–26)	0.7 (0.2–2.2)	1.8 (0.5–6.6)	5.4	3.4 (0.9–13.1)

AOPSS, Ambulatory Oncology Patient Satisfaction Survey; YVM, Your Voice Matters survey; OR, Odds Ratio; CI, Confidence Interval; GI, Gastrointestinal; and GU, Genitourinary. OR < 1 means worse experience; OR > 1 means better experience, OR (in bold font) represents a statically significant difference. Due to the sample size and structure of our data, quasi-complete separations occurred in a few models, where a level within a covariate has completely “empty” cells, causing the parameter estimates to become infinite in size, which we reported as “NA”. The model’s other covariates remain unaffected and are reported.

## Data Availability

No new data were created or analyzed in this study. Data sharing is not applicable to this article.
